# Statistical distribution of blood serotonin as a predictor of early autistic brain abnormalities

**DOI:** 10.1186/1742-4682-2-27

**Published:** 2005-07-19

**Authors:** Skirmantas Janušonis

**Affiliations:** 1 Yale University School of Medicine, Department of Neurobiology, P.O. Box 208001, New Haven, CT 06520-8001, USA

## Abstract

**Background:**

A wide range of abnormalities has been reported in autistic brains, but these abnormalities may be the result of an earlier underlying developmental alteration that may no longer be evident by the time autism is diagnosed. The most consistent biological finding in autistic individuals has been their statistically elevated levels of 5-hydroxytryptamine (5-HT, serotonin) in blood platelets (platelet hyperserotonemia). The early developmental alteration of the autistic brain and the autistic platelet hyperserotonemia may be caused by the same biological factor expressed in the brain and outside the brain, respectively. Unlike the brain, blood platelets are short-lived and continue to be produced throughout the life span, suggesting that this factor may continue to operate outside the brain years after the brain is formed. The statistical distributions of the platelet 5-HT levels in normal and autistic groups have characteristic features and may contain information about the nature of this yet unidentified factor.

**Results:**

The identity of this factor was studied by using a novel, quantitative approach that was applied to published distributions of the platelet 5-HT levels in normal and autistic groups. It was shown that the published data are consistent with the hypothesis that a factor that interferes with brain development in autism may also regulate the release of 5-HT from gut enterochromaffin cells. Numerical analysis revealed that this factor may be non-functional in autistic individuals.

**Conclusion:**

At least some biological factors, the abnormal function of which leads to the development of the autistic brain, may regulate the release of 5-HT from the gut years after birth. If the present model is correct, it will allow future efforts to be focused on a limited number of gene candidates, some of which have not been suspected to be involved in autism (such as the 5-HT_4 _receptor gene) based on currently available clinical and experimental studies.

## Background

Our ability to treat and prevent autism is severely limited by our lack of knowledge of what biological abnormality causes this developmental disorder. Since autism is considered primarily a brain disorder, much of the research over the past decades has focused on the autistic brain. Different groups have reported a wide range of anatomical abnormalities in autistic brains, such as reduced numbers of Purkinje cells in the cerebellum [1-3]; an unusually rapid growth of the cerebral cortical volume and head circumference during the first years after birth [4-9]; abnormal cortical minicolumns [10-13]; abnormalities of the limbic system [14-19]; abnormalities of the brainstem [20-22]; and other brain alterations [23-25].

Considering the complexity of brain development and its highly dynamic nature, these abnormalities may be the result of a long, complex chain of events. The original abnormality that caused them may occur early in development [26] and may be no longer obvious by the time autism is diagnosed. For example, an autistic-like loss of Purkinje cells may be caused by a mutation of the *toppler *gene, which causes severe ataxia in mice and appears to be irrelevant to autism [27]. *Post-mortem *analysis of younger autistic brains is not an option, because it is usually not clear until age 2 or 3 which brains are autistic and which are not.

Fortunately, evidence suggests that at least one biological factor that causes the development of the autistic brain has a different function outside the central nervous system (CNS), where it continues to operate well into childhood and perhaps even into adulthood. Since the early 1960s, the most consistent biological finding in autistic individuals has been their statistically elevated serotonin (5-hydroxytryptamine, 5-HT) levels in blood platelets, or platelet hyperserotonemia [28-33]. Unlike many of the reported alterations in the brain, this finding has been replicated numerous times by different groups, some of which have used large numbers of subjects. According to Anderson [33], "the platelet hyperserotonemia of autism [...] is generally considered to be one of the more robust and well-replicated findings in biological psychiatry". The main reason why we have not capitalized on this major finding is that we have not been able to understand its origin or its relation to the brain.

It is unlikely that the autistic platelet hyperserotonemia is induced by the brain. The human blood-brain barrier (BBB) becomes mature around one year after birth, if not earlier [34,35], and is virtually impenetrable to 5-HT. Tryptophan, a 5-HT precursor, can cross the BBB, but tryptophan levels do not appear to be altered in autistic individuals [36]. Unlike the anatomy of the mature brain, platelet 5-HT levels should be actively maintained, because the half-life of platelets is only a few days [37,38]. This suggests that the factor that causes the platelet hyperserotonemia continues to be functionally active years after birth.

**Figure 1:**
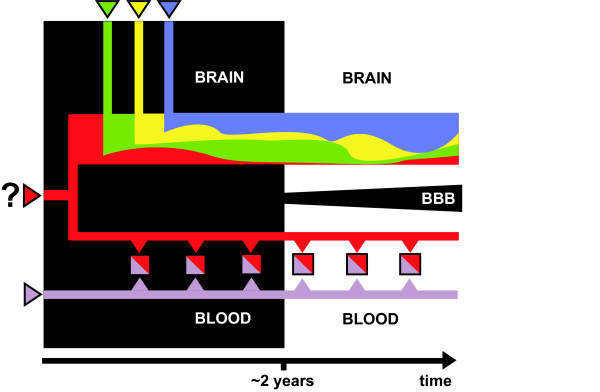
**A biological factor that causes autism may have a dual function**. A factor that causes autism (shown in red) may be expressed (1) in the CNS, where it plays a role in the early development of the brain, and (2) outside the CNS, where it participates in processes that determine the 5-HT levels in blood platelets. The "central" and "peripheral" 5-HT systems are separated by the blood-brain barrier (BBB) that matures after birth. It is usually not clear until age 2 or 3 whether the brain is autistic (black box). By that time, the factor has altered numerous developmental processes in the brain and may no longer be obvious. This same factor continues to operate years after birth outside the CNS, where it maintains higher than normal 5-HT levels in blood platelets. In contrast to the brain, blood platelets are short-lived and continue to be produced throughout the life span.

The statistical distribution of platelet 5-HT levels in normal and autistic groups has certain characteristic features [31], but only recent studies have attempted to describe them in detail [39,40]. These distributions are likely to contain information about the underlying processes controlling platelet 5-HT levels and, therefore, may help identify the factor that causes the platelet hyperserotonemia of autism. This same biological factor may be active during brain development (not necessarily in the same role), but there its identity may be obscured by the final complexity of a several-year-old autistic brain (Fig. 1). In the present study, published distributions of blood 5-HT levels are analyzed by a novel, quantitative approach that may help trace early, experimentally undetectable brain abnormalities leading to autism.

## Results

### Basic model

The origin of the platelet hyperserotonemia of autism cannot be understood unless a certain model of the underlying physiological processes is accepted – whether it is an implicit model that is not clearly stated, a model described in words, or a mathematical model. One advantage of mathematical modeling is that it requires a clear description of all relevant interactions among the components of the system. Its greatest disadvantage is that sometimes clear-cut choices have to be made where experimental data may suggest a few possible alternatives. In this section I introduce a model that is based on what is known about the 5-HT circulation outside the CNS and point out two important but unresolved problems.

In search of a factor that can both cause platelet hyperserotonemia and alter normal brain function, many recent studies have focused on the serotonin transporter (SERT) that is expressed in blood platelets and brain neurons [41]. Despite early promising results [42], different groups have found little or no linkage [43] between SERT polymorphisms and autism in various ethnic groups [40,44-47]. I have recently proposed [48] that the factor that interferes with brain development in autism may also regulate the release of 5-HT from gut enterochromaffin (EC) cells, the main source of blood 5-HT [36,49,50]. First, this hypothesis assumes that EC cells can monitor (directly or by way of gastrointestinal neurons) the 5-HT levels in the surrounding extracellular space and can decrease or increase their 5-HT release accordingly. Similar control mechanisms have long been suspected in the brain, where serotonergic neurons express 5-HT autoreceptors [51,52]. Second, the levels of extracellular 5-HT in the gut wall are assumed to be at equilibrium with the levels of free 5-HT in the arterial blood. While the baseline extracellular levels of 5-HT in the gut wall have not been precisely measured, the estimated levels of free 5-HT in the arterial blood appear to be comparable to the extracellular 5-HT levels in the brain [51,53], which expresses some of the same 5-HT receptors as the gut [51,54-57].

This hypothesis can be cast in a mathematical form. Suppose that EC cells indirectly monitor the levels of free 5-HT that arrives in the gut with the arterial blood, compare these levels with the expected 5-HT levels, and adjust their 5-HT release to a new value (*R*_*n*+1_), using a pre-set release value (*R*_*C*_) as the reference point. The strength (gain) of this adjustment is controlled by a factor *α*, which is hypothesized to be different in normal and autistic individuals. After the blood leaves the gut, a large proportion (*γ*) of the free 5-HT is quickly removed by the liver, lungs and other organs that express SERT and monoamine oxidases (MAOs) [58-62]. The numerical value of *γ *is likely to vary from individual to individual, because the SERT and MAO genes have a number of polymorphic variants distributed in the population [40,45,46,63-66]. Therefore, *γ *is considered to be a random variable with a known probability distribution. The model can then be described by the following system of equations:



*F*_*n *+ 1 _= (1 - *γ*)*F*_*n *_+ *R*_*n *+ 1_,     (2)

Where (1 - *γ*)*F*_*n *_is the flux of free 5-HT that enters the gut with the arterial blood, *F*_*C *_is the pre-set ("expected") flux, and *F*_*n *+ 1 _is the flux of free 5-HT that exits the gut (α ≥ 0, 0 ≤ γ ≤ 1, *F*_*C *_> 0, *R*_*C *_> 0). In the model, the 5-HT release from EC cells does not include the 5-HT that is used for local signaling and is rapidly removed by local gastrointestinal epithelial and neural cells expressing SERT [54,67,68]. This 5-HT could be included in the model, together with the local clearance rate, if estimates of these parameters were available.

It is thought that little free 5-HT is taken up by blood platelets, before most of it is removed by the liver, lungs and other organs [53,60]. Also, it has been suggested that platelet 5-HT levels may depend on the levels of free 5-HT in the blood almost linearly [53]. Then, at the steady state, *F*_*n *+ 1 _= *F*_*n *_≡ *F *and *R*_*n *+ 1 _= *R*_*n *_≡ *R *for any *n*, and platelet 5-HT levels are



where *K *> 0 is a constant.

Note that *ser*(*α*, *γ*) is a decreasing function of *γ*. Also, at the steady state,

*R *= *γF*.     (4)

It should be emphasized that the mathematical simplicity of equations (1) and (2) in no way implies that the biological regulation of 5-HT release in the gut is simple. The human gut is a remarkably complex organ that uses a wide range of neurotransmitters and that may have at least as many neurons as the spinal cord [50]. Nevertheless, recent studies suggest that complex biological systems, such as brain neurons, can be "actively linear" [69], meaning that sophisticated biological mechanisms may act on intrinsically non-linear physical processes to produce quantitative relationships that are mathematically linear.

**Figure 2:**
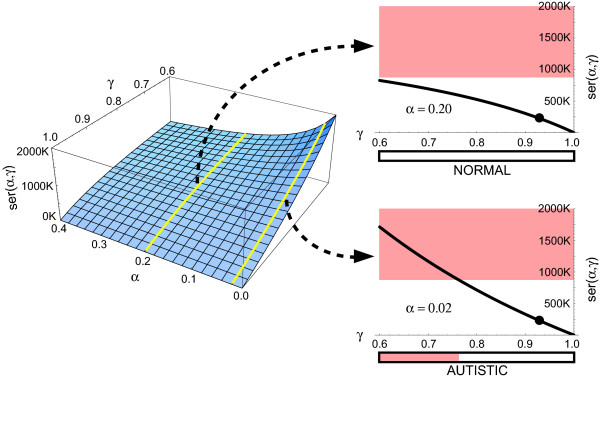
**Platelet levels as a function of *α *and *γ***. Platelet 5-HT levels, *ser*(*α*, *γ*), plotted as a function of *α *(the factor regulating 5-HT release from EC cells) and *γ *(the rate of 5-HT clearance by the liver, lungs, and other organs). This relationship is described by equation (3), where *K *is a constant. Note that if *α *is normal (high), platelet 5-HT levels stay low with any *γ*, but if *α *is autistic (low), individuals with a low *γ *become hyperserotonemic. The black circles mark the points whose coordinates are independent of *α *and are γ* = *R*_*C*_/(*R*_*C *_+ *F*_*C*_) and *ser*(*α*, γ*) = *KF*_*C*_. Note in equations (1) and (2) that *R *= *R*_*C *_if and only if *γ *= γ*, so the distribution of *γ *is likely to contain γ*. This guarantees that the distributions of the 5-HT levels in normal autistic groups will always overlap, as observed in clinical studies. For illustrative purposes, the normal and autistic values of *α *were arbitrarily set at 0.20 and 0.02, respectively. These are realistic values, as follows in the text. The other parameter values were taken from published studies [48, 53, 70] and were *F*_*C *_= 210 ng/min and *R*_*C *_= 3000 ng/min.

The dependence of platelet 5-HT levels on *α *and *γ *is plotted in Figure 2, where the numerical values of *F*_*C *_and *R*_*C *_are taken from previously published experimental and theoretical studies [48,53,70], and where the regulation of the 5-HT release from EC cells is assumed to be less than fully functional in autistic individuals (note the low *α *value). A key feature of this dependence is that, in normal individuals, platelet 5-HT levels remain low with any *γ*, whereas in autistic individuals these levels may be normal or higher than normal depending on the individual's *γ*. This dependence captures one of the most puzzling properties of the autistic distribution of platelet 5-HT levels, which always overlaps with the control (normal) distribution, but always includes individuals whose 5-HT levels are higher than normal [31]. It may also explain why the SERT and MAO genes may appear to be linked with autism but may not actually cause it. As shown in Figure 2, a low *γ *is a necessary but not sufficient condition for the platelet hyperserotonemia to occur. Given a low *γ*, the platelet hyperserotonemia will occur only in those individuals whose regulation of the 5-HT release from EC cells is compromised (i.e., they are autistic and have a low *α*). It follows then that *γ *acts only as a modifier of platelet 5-HT levels, and that the statistical distribution of *γ *may be the same in normal and autistic populations. Assuming an individual's *γ *value is determined, at least in part, by his/her variants of the SERT and MAO genes expressed in the liver, lungs and other organs, normal and autistic populations may have similar distributions of SERT and MAO polymorphisms. This assumption is supported by recent studies [40,45-47,63,64].

Two potentially contentious decisions were made in the model. First, the exact levels of free 5-HT in the blood remain a debated issue. While a number of studies have found "low" but consistently measurable levels of free 5-HT in the human blood [53,70,71], Chen et al. [72] have suggested that the concentration of free 5-HT in the blood may be negligible, since these researchers have detected virtually no 5-HT in the whole blood of SERT-deficient mice whose blood platelets cannot take up 5-HT. Second, the model assumes that virtually all of the 5-HT stored in blood platelets is taken up by them after the lungs, liver, and other organs have cleared a large proportion of the 5-HT released by the gut. While evidence exists this may be the case [53,60], not all researchers agree. One could conceivably take into account both of these views by setting

*ser*(*α*, *γ*) ≡ *K*_1 _*F *+ *K*_2_(1 - *γ*)*F*

or, in a more general form,



where *K*_1_, *K*_2 _≥ 0 are constants and *K*(*ω*) is a function. However, this would require more detailed information about the dynamics of the 5-HT uptake by platelets, which is not currently available [31].

### Distributions generated by the model

While the model (Fig. 2) appears to capture some of the key characteristics of the reported platelet 5-HT levels, it remains unclear whether it would produce similar results if *α *and *γ *took on other numerical values. The regulation of the 5-HT release in EC cells is poorly understood and no experimental estimates for the parameter *α *are available. Is it actually lower in autistic individuals? Likewise, how reasonable is it to suppose that the distribution of *γ *is the same in normal and autistic groups? Importantly, would the model produce consistent numerical values of parameters if different experimental studies were used?

To answer these questions, one may consider the basic framework of the model to be correct, but make no *a priori *assumptions about the values of the parameters (with the exception of those that are experimentally known) or about their differences in normal and autistic individuals. Then the unknown parameters of the model may be allowed to vary in the numerical space until the statistical distributions of 5-HT levels produced by the model closely match those reported in actual clinical studies. In order to be able to do this, one first has to find the theoretical statistical distributions of platelet 5-HT levels produced by the model.

The exact population distribution of *γ *is unknown, but its mean value is likely to be close to one [60]. Since SERT gene polymorphisms may occur with comparable frequencies [73], the statistical distribution of *γ *in a population can be approximated by a continuous uniform distribution on the interval [*a*, *b*] with the probability density function



It can be shown from equations (3) and (5) that the probability density function of platelet 5-HT levels then is



The theoretical population mean *μ*_*ser*_(*α*, *a*, *b*) and variance (*α*, *a*, *b*) of platelet 5-HT levels follow immediately:



and



where *U *≡ *F*_*C *_- *R*_*C*_*α*.

The standard deviation of platelet 5-HT levels in the population then is



### Distributions reported in clinical studies

Mean values of normal and autistic blood 5-HT levels have been reported and discussed in numerous publications [28-33]. In contrast, the precise statistical distributions of the platelet 5-HT levels in normal and autistic groups, such as their histograms (which roughly approximate their theoretical probability density functions), have so far attracted little attention. Only a few recent reports have presented more detail about the shape of these distributions. These reports are used in the following analysis:

(i) *Mulder et al. *[39] is recent and perhaps the most reliable report to date. It has used a relatively large sample of subjects whose platelet 5-HT levels are presented in histograms. The authors of this report are well-established researchers of blood 5-HT and autism. One of the co-authors, G.M. Anderson, has had numerous publications on the subject over the past several decades.

(ii) *Coutinho et al. *[40] have studied a large sample of subjects and presented their 5-HT levels in histograms, also explicitly listing their minimum and maximum values. However, their reported mean 5-HT levels are somewhat low, and the autistic 5-HT levels are higher than, but not significantly different from, the normal 5-HT levels.

(iii) *McBride et al. *[74] is a detailed report on the means and standard variations of platelet 5-HT levels in ethnically different groups, but the data are not presented in histogram form. Here, the minimum and maximum values of the distributions are recovered from their Figure 1, and the pooled means of the pre-pubertal children are recalculated from their Table 2.

**Table 2:** Predicted and observed ranges, means (<*ser*>), and standard deviations (*SD*) of platelet 5-HT levels, *ser*(*α*, *γ*). The distribution of *γ *was assumed to be continuously uniform; the theoretical *SD *values given in the table can be further improved by assuming that *γ *has a beta distribution or a normal distribution (see the text). Note that, strictly speaking, the model's <*ser *> and *SD *are precise theoretical expectations and standard deviations and, therefore, the notation *μ*_*ser *_(*α*, *a*, *b*) and *σ*_*ser *_(*α*, *a*, *b*) would be more accurate (but less convenient here).

	**Mulder et al. [39] **(nmol/10^9 ^platelets)		**Coutinho et al. [40] **(ng/10^9 ^platelets)		**McBride et al. [74] **(ng/ml)	
	Model	Observed	Model	Observed	Model	Observed

						
***Min***_normal_	1.42	0.67	0	66	75	85
***Max***_normal_	5.57	5.67	598	676	417	449
**<*ser*>**_normal_	3.66	3.58	320	260	252	230
***SD***_normal_	*1.19*	*1.08*	*172*	*137*	*99*	-
						
***Min***_autistic_	1.37	2.33	0	50	73	120
***Max***_autistic_	8.18	8.33	925	1125	546	567
**<*ser*>**_autistic_	4.58	4.51	414	304	294	287
***SD***_autistic_	*1.96*	*1.61*	*265*	*207*	*136*	-

It is important to note that these reports are the only ones presently available and, therefore, no selection bias was introduced by choosing them for the present study.

### Finding *α *and [*a*, *b*] from clinical data

In order to be able to compare the model's predictions with actual clinical reports, the numerical output of the model has to be scaled to the units of the used experimental studies. This scaling can be done by adjusting the parameter *K *in equation (3). The studies have reported the following means of the blood 5-HT levels in their normal groups: 3.58 nmol/10^9 ^platelets [39], 260 ng/10^9 ^platelets [40], and 230 ng/ml [74]. The last number was obtained by pooling the reported pre-pubertal means of the three ethnic groups. Assuming the flux of free 5-HT to the gut is around 210 ng/min in normal individuals [48,53,70], it follows from equation (3) that



where <...> denotes experimentally obtained means. Now we can calculate the approximate *K *values for each of the studies by dividing their reported mean 5-HT levels by the approximate flux of free 5-HT to the gut. This yields the following *K *values for the reports of Mulder et al. [39], Coutinho et al. [40] and McBride et al. [74], respectively: 0.0170 (nmol min ng^-1 ^10^-9 ^platelets), 1.2381 (min 10^-9 ^platelets), and 1.0952 (min ml^-1^).

Next, we try to find such numerical values of [*a*, *b*], *α*_*normal*_, and *α*_*autistic*_, that they minimize the difference between the predicted and observed levels of blood 5-HT. Suppose that the observed levels of blood 5-HT vary from *Min*^OBS ^to *Max*^OBS ^and that the observed mean of blood 5-HT is <*ser*>^OBS^. The following error function can then be constructed:



where







and *i *= *normal*, *autistic*.

Note that, compared with the mismatch between the predicted and observed ranges of the distributions, the mismatch between the predicted and observed means is penalized "twice as much", because observed means are likely to be more accurate than observed minimal and maximal values.

This error function was numerically minimized by using the standard Nelder-Mead (downhill simplex) and differential evolution methods [75] implemented in Mathematica's *NMinimize *function (Wolfram Research, Inc.). Since the values of *R*_*C *_and *F*_*C *_may be approximated from published studies but are not necessarily accurate, *R*_*C *_was centered at 3000 ng/min based on a published estimate [53] and was allowed to vary ± 33%, whereas the value of *F*_*C *_was centered at 210 ng/min based on published estimates [48,53,70] and was allowed to vary ± 50% (more variation was allowed for *F*_*C *_because less is known about its actual value). No constraints were set for the interval [*a*, *b*] (i.e., 0 ≤ *a *<*b *≤ 1). The variables *α*_*normal *_and *α*_*autistic *_were allowed to vary from 0 to 5 and no *a priori *assumptions were made about their relative values (i.e., both *α*_*normal *_>*α*_*autistic *_and *α*_*normal *_≤ *α*_*autistic *_were allowed). It can be shown that the system (equations (1) and (2)) is stable if *0≤α<F*_*C*_(2 - *γ*)/[R_*C*_(1 - *γ*)]. Since the system should be stable for any γ ∈[*a*, *b*] and [*a*, *b*] is likely to contain the point *γ *≈ 0.99 [60] or *γ *≈ 0.93 [48], choosing *α *between 0 and 5 allows the optimization procedure to use virtually any value of *α *where the system maintains stability.

The numerical values of the model's parameters (*α*_*normal*_, *α*_*autistic*_, [*a*, *b*], *F*_*C*_, and *R*_*C*_) that minimized the error function are given in Table 1. Note that all three clinical studies yielded similar sets of values. Most importantly, the minimization algorithms yielded the best match between the model and the clinical reports when *α*_*autistic *_was virtually zero.

**Table 1:** Estimates of *F*_*C*_, *R*_*C*_, *a*, *b*, *α*_*normal*_, and *α*_*autistic*_, obtained by numerical minimization of the error function.

Data source	*K*	*F*_*C*_	*R*_*C*_	*a*	*b*	*α*_*normal*_	*α*_*autistic*_
Mulder et al. [39]	0.0170	105	2000	0.8060	0.9612	0.1510	0.0000
Coutinho et al. [40]	1.2381	105	2000	0.7280	1.0000	0.0981	0.0000
McBride et al. [74]	1.0952	105	2000	0.8006	0.9678	0.0895	0.0000

By plugging these obtained values of the parameters into equations (12), (13), (14) and (9), one can obtain the values of 5-HT levels predicted by the model and compare them with the actual observed levels. As shown in Table 2, the predicted values closely match the values observed in Mulder et al. [39] and McBride et al. [74]. The largest mismatch was between the predicted and observed minimal values. The model predicted slightly higher mean 5-HT levels for Coutinho et al. [40] than were actually observed; interestingly, Coutinho et al. [40] have in fact reported unusually low platelet 5-HT levels.

### Distribution of *γ *can be approximated by beta and normal distributions

**Figure 4:**
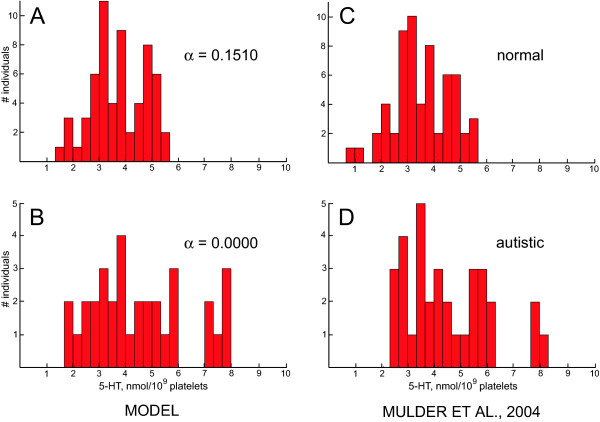
**Model replicates published data**. ***A***, ***B***, The model's simulation of Mulder et al.'s sampling [39], assuming *γ *has the beta distribution on the interval [0.8060, 0.9612] with both shape parameters equal to 1.5. The platelet 5-HT levels were calculated by using equation (3), with the values of *K*, *F*_*C*_, *R*_*C*_, *α*_*normal *_and *α*_*autistic *_taken from Table 1. ***C***, ***D***, The actual data from Mulder et al. [39] (reprinted by permission from Lippincott Williams & Wilkins, modified). In the simulated and actual sampling, 60 normal and 33 autistic subjects were used. Note that the exact appearance of the histograms will vary from sampling to sampling due to the small number of cases in each bin.

**Figure 5:**
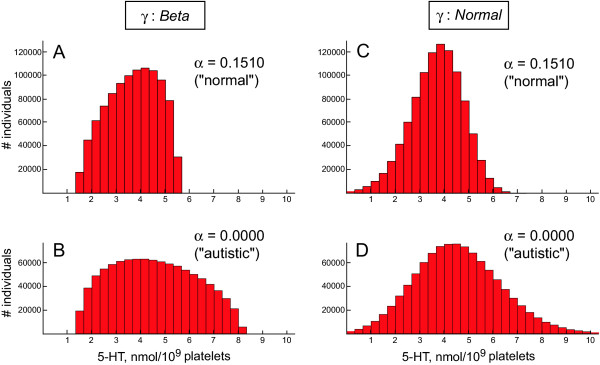
**Model predicts the shape of the normal and autistic distributions of platelet 5-HT levels**. Histograms obtained by simulating a sampling of a very large number of normal and autistic individuals (a million subjects in each group). The distribution of *γ *was assumed to be (***A***, ***B***) the beta distribution on the interval [0.8060; 0.9612] with both shape parameters equal to 1.5 (see the text); or (***C***, ***D***) the normal (Gaussian) distribution with mean 0.8836 (the midpoint of the interval [0.8060; 0.9612]) and standard deviation 0.04 (see the text). The platelet 5-HT levels were calculated by using equation (3), with the values of *K*, *F*_*C*_, *R*_*C*_, *α*_*normal *_and *α*_*autistic *_taken from Table 1. In a very large sampling, the number of cases in each histogram bin closely approximates the number of cases predicted by the exact probability distribution functions. The Chi-square test confirmed that the normal and autistic distributions predicted by the model may underlie the distributions reported by Mulder et al. (2004). The following goodness-of-fit results were obtained:  = 12.38 (*P *= 0.26) and  = 11.29 (*P *= 0.19) for the normal and autistic groups, respectively, if *γ *had the beta distribution; and  = 13.36 (*P *= 0.27) and  = 12.21 (*P *= 0.14) for the normal and autistic groups, respectively, if *γ *had the normal distribution (bins were pooled if theoretical bins had fewer than 3 cases). It is important that both the normal and autistic distributions had the same underlying distribution of *γ *and that only one parameter, *α*, was needed to switch from the normal distribution to the autistic distribution. Also, compare the histograms in *C *and *D*, based on the data of Mulder et al. [39], with those in Figure 1 of Coutinho et al. [40].

One advantage of choosing the uniform distribution to represent *γ *is that it simplifies calculations and allows finding the exact formulae for means and standard deviations. However, the model tends to overestimate the standard deviations of platelet 5-HT levels (Table 2), because in the uniform distribution even extreme *γ *values occur with same probability as all others. Instead of approximating the distribution of *γ *as uniform, one may want a distribution of which the probability density function drops off more smoothly near the minimal and maximal values. This can be achieved by replacing the uniform distribution of *γ *with the beta distribution, the uniform distribution being its special case [76]. The following deals with mathematical technicalities of this replacement. Non-mathematically inclined readers may skip them and go immediately to Figures 4 and 5 referred to at the end of this section.

**Figure 3:**
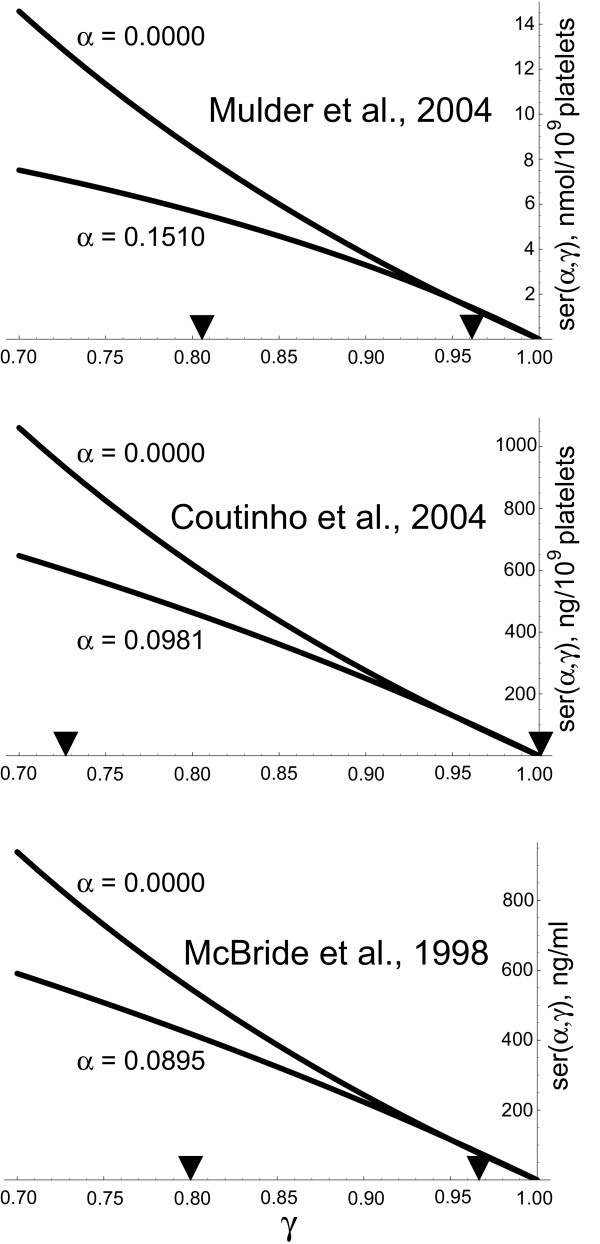
**Platelet levels plotted with the parameter values derived from published studies**. Platelet 5-HT levels as functions of *γ *for the data of Mulder et al. [39], Coutinho et al. [40] and McBride et al. [74]. Equation (3) and the estimated parameter values from Table 1 were used. The arrowheads mark the predicted intervals of the *γ *distributions (Table 1). For comparison, the Y-axes were scaled proportionally to the *K *values of the three studies (Table 1).



Note that if the obtained parameter values (Table 1) are plugged into equation (3), the normal and autistic platelet 5-HT levels turn out to depend on *γ *almost linearly (Fig. 3). This allows "warping" the uniform distribution of *γ *into a symmetric beta distribution on the same interval, with little effect on the theoretical mean values of *ser*(*α*, *γ*). Suppose that *γ *has a symmetric beta distribution on [*a*, *b*], whose shape is determined by the parameters *m *and *n*, such that *m *= *n *(if *m *= *n *= 1, the beta distribution becomes the uniform distribution). We can use a Taylor series to formally linearize *ser*(*α*, *γ*) around *γ*_0 _= (*a *+ *b*)/2 as *ser*(*α*, *γ*) ≈ *ser*(*α*, *γ*_0_) - *λ *(*γ *- *γ*_0_) ≡ *serL*(*α*, *γ*),

Then, keeping in mind that *γ *has a beta distribution, the standard deviation of *serL*(*α*, *γ*) becomes



Since the values of *λ*, *a*, and *b *have already been estimated (Table 1), it is now possible to obtain the *m *values that yield such standard deviations of the linearized *ser*(*α*, *γ*) that they precisely match those reported in the clinical studies (Table 2). The following *m *values were obtained for the normal and autistic groups, respectively: 1.2940 and 1.7028 for the data of Mulder et al. [39]; and 1.8308 and 1.8748 for the data of Coutinho et al. [40]. Pooled standard variations were unavailable in McBride et al. [74]. We have earlier assumed that normal and autistic groups have the same *γ *distribution. Therefore, the actual *m *values can be approximated by 1.50 for Mulder et al. [39] and 1.85 for Coutinho et al. [40].

Likewise, *γ *can be assumed to have a normal distribution with mean (*a *+ *b*)/2 and standard deviation *σ*. Then the standard deviation of *serL*(*α*, *γ*) becomes

*σ*_*serL*_(*α*, *a*, *b*, *σ*) = *λσ*,     (17)

where *λ *is the same as in equation (15), and we obtain the following *σ *values for the normal and autistic groups, respectively: 0.0410 and 0.0370 for the data of Mulder et al. [39]; and 0.0630 and 0.0624 for the data of Coutinho et al. [40]. Therefore the actual *σ *values can be approximated by 0.04 for Mulder et al. [39] and 0.06 for Coutinho et al. [40].

The model now easily generates "normal" and "autistic" samples of platelet 5-HT levels that closely match the actual reported data (Fig. 4). Most importantly, the switch from the normal distribution to the autistic distribution requires changing only one parameter, *α*.

It is not known what normal and autistic distributions would look like if one could sample a very large number of subjects. The model can predict the shape of these distributions by simulating such large sampling (Fig. 5).

### Is the 5-HT synthesis rate altered in autism?

One of the most important questions in autism research is whether the rate of 5-HT synthesis is altered in the brain and gut of autistic individuals. If 5-HT synthesis is altered in the autistic brain, as some studies have suggested [77-79], this potentially may have a great impact on brain development [80,81] (but caution should be exercised in predicting the extent of these alterations [82]).

The brain 5-HT and the gut 5-HT are synthesized by two different tryptophan hydroxylases [49] that, at least in humans, have different properties and are regulated differently [83]. While the biological factor underlying the parameter *α *of the model is hypothesized to play a role in the developing brain (Fig. 1), the model makes no assumptions about its exact function in the brain. In the brain, it may not regulate 5-HT release from serotonergic neurons and may have a different function (see, for example, Figure 4 of [48]). Therefore, this section focuses only on the 5-HT synthesis and release in the gut.

It is important to note that the model says nothing about the rate of 5-HT synthesis in the gut and rather deals with the rate of 5-HT release from the gut. However, most clinical and experimental studies make no such distinction and, therefore, their relevance to the model is discussed assuming higher 5-HT synthesis rates do lead to higher 5-HT release rates.

It follows from equations (3) and (4) that, at the steady state,



and that this relationship is independent of *γ*. This means that if one were to sample any group of individuals and could measure their platelet 5-HT levels and gut 5-HT release rates precisely, the correlation coefficient between these two variables would always be minus one, irrespective of the distribution of *γ*. In other words, equation (18) predicts that individuals with higher platelet 5-HT levels should have lower 5-HT release rates.

How can lower 5-HT release rates lead to higher platelet 5-HT levels? Note that, in the model, both the platelet 5-HT levels and the 5-HT release rate are dynamically linked through the 5-HT clearance rate, *γ*. As *γ *grows lower, less 5-HT is removed from the system and more of 5-HT is accumulated in blood platelets. At the same time, these higher 5-HT levels drive down the 5-HT release rate in the gut, as required by equation (1).

Still, it appears that the results of clinical studies are inconsistent with equation (18). Three important findings should be noted:

(i) Minderaa et al. [36] have found no significant correlation between whole blood 5-HT levels and 5-HT synthesis in the gut, measured as the production of urinary 5-HIAA [36]. Similar results have been obtained by Launay et al. [84] and other groups (reviewed in [31]).

(ii) Croonenberghs et al. [85] have shown that the 5-HT synthesis in the gut of autistic individuals may be higher than that in normal individuals, at least when subjects are administered 5-hydroxytryptophan (5-HTP), an immediate precursor of 5-HT.

(iii) Carcinoid tumors, derived from gut EC cells, may result in excessive synthesis and release of 5-HT, which in turn may lead to elevated platelet 5-HT levels [86].

A more careful analysis reveals that these findings are not only consistent with the model, but that the model can reconcile some of the apparent contradictions among them:

(i) It follows from the model that the measured correlation between platelet 5-HT levels and 5-HT release rates should be close to zero in autistic groups, even though equation (18) holds.

In fact, we can rewrite equation (18) as



Now consider two random variables, *η *and *ξ*, that are linearly dependent such that

*η *= *wξ *+ *q*,     (20)

where *w *and *q *are constants. It follows from equation (20) that the correlation between them is either -1 or 1, depending on the sign of *w*.

Denote the means of these variables *μ*_*η *_and *μ*_*ξ*_, respectively, and their standard deviations *σ*_*η *_and *σ*_*ξ*_, respectively. Suppose next that the errors of measurement of *η *and *ξ *are independent random variables *ε*_*η *_and *ε*_*ξ*_, such that their expected values are zero and standard deviations are *δ*_*η *_and *δ*_*ξ*_, respectively. Note that experimentally we can measure only η* = *η *+ *ε*_*η *_and ξ* = *ξ *+ *ε*_*ξ*_. The expected values of η* and ξ* are the same as those of *η *and *ξ*. However, the theoretical correlation coefficient between η* and ξ* now becomes



If the standard deviations of the errors of measurement are small, we obtain *ρ*(η*, ξ*) ≈ ± 1, as expected from equation (20).

Now we return to equation (19). Any experimental measurement of *R *(5-HT release) and *ser*(*α*, *γ*) (platelet 5-HT levels) will contain a measurement error. Denoting these measured values ser*(*α*, *γ*) and R*, one obtains from equations (19), (20), and (21) that the correlation coefficient between R* and ser*(*α*, *γ*) is



where

*w *= -(*αR*_*C*_)/(*KF*_*C*_),     (23)

*σ*_*ser *_> 0 is the standard deviation of *ser*(*α*, *γ*), and δ_R_ > 0 and δ_ser_ > 0 are the standard deviations of the errors of measurement of *R *and *ser*(*α*, *γ*), respectively. The estimated values of *K*, *F*_*C*_, *R*_*C*_, and *α *can be obtained from Table 1 and the values of *σ*_*ser *_from Table 2 or from the original published data.

Consider now an autistic group whose *α *→ 0 (Table 1). Then, from equation (23), *w *→ 0, and it follows from equation (22) that .

(ii) Croonenberghs et al. [85] have recently shown that oral administration of 5-hydroxytryptophan (5-HTP) leads to higher platelet 5-HT levels in autistic patients, and the authors have suggested that the 5-HT synthesis rate may be higher in the gut of autistic subjects compared with normal subjects.

Suppose that the administered 5-HTP is converted to 5-HT at the same rate in both normal and autistic groups. It is likely that the exogenous influx of 5-HTP results in a comparable exogenous influx of 5-HT, because the rate-limiting step in the synthesis of 5-HT is not the 5-HTP conversion to 5-HT, but rather the tryptophan conversion to 5-HTP [87].

Notice that the system is not in its steady state during the experiment and, therefore, we have to use equations (1) and (2), which now should contain the exogenous source of 5-HT. It is straightforward to see that the system then becomes



*F*_*n *+ 1 _= (1 - *γ*)*F*_*n *_+ *R*_*n *+ 1 _+ *R*_*EX*_,     (25)

where *R*_*EX *_is the exogenous flux of 5-HT.

**Figure 6:**
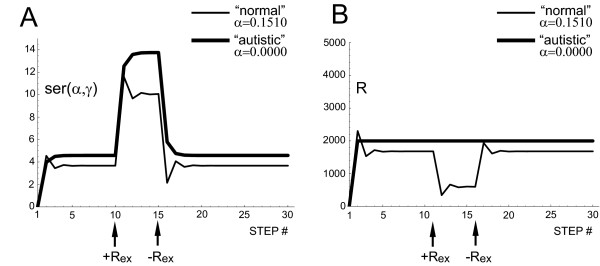
**An exogenous source of 5-HT elevates platelet 5-HT levels in an autistic group more than in a normal group**. For the simulation, the initial values of platelet 5-HT levels (*ser*(*α*, *γ*)) and 5-HT release rate (*R*) were set at zero and the system developed according to equations (1) and (2). After the system reached its steady state, an exogenous 5-HT source was "turned on" (+*R*_*EX*_) and the system developed according to equations (24) and (25). After 5 steps, the exogenous 5-HT source was "turned off" (-*R*_*EX*_) and the system developed according to equations (1) and (2) until it returned to its steady state. Each point is the mean of 10,000 simulated individuals whose *γ *had the beta distribution on the interval [0.8060, 0.9612] (see Table 1) with both shape parameters equal to 1.5 (see the text). Individual plots (not shown) looked essentially the same as the mean plots. The ratio between the autistic and normal platelet 5-HT levels (*A*) at step 7 (at the steady state) is 1.25 and the same ratio at step 13 is 1.35. The numerical values of the parameters were *K *= 0.0170, *F*_*C *_= 105, *R*_*C *_= 2000, *α*_*normal *_= 0.1510, *α*_*autistic *_= 0.0000 (Table 1) and *R*_*EX *_= 4000. Compare these plots with Figure 1 of Croonenberghs et al. [85].

Solving equations (24) and (25) step-by-step essentially replicates the major finding of Croonenberghs et al. [85] (Fig. 6). However, the model predicts that the higher blood 5-HT levels in autistic subjects are not due to a higher 5-HT synthesis rate, but rather to the failure of their gut to decrease the release of endogenous 5-HT in response to the high concentration of 5-HT caused by the administration of 5-HTP.

(iii) In the case of carcinoid tumors, abnormally large amounts of 5-HT may be released into the blood. It is likely that the normal mechanisms regulating 5-HT release are compromised or absent in carcinoid tumors. Then instead of equations (1) and (2) one can consider only one equation (2), which can be rewritten as

*F*_*n *+ 1 _= (1 - *γ*)*F*_*n *_+ *R*_*CARCINOID*_,     (26)

where *R*_*CARCINOID *_is large and relatively constant. Then, at the steady state,

*F *= *R*_*CARCINOID*_/*γ*

and



It is obvious that in this abnormal case higher 5-HT release rates will lead to higher platelet 5-HT levels, as reported by Kema et al. [86].

## Discussion

The presented model is based on the hypothesis that at least one factor that interferes with normal brain development in autism also participates in the regulation of 5-HT release from enterochromaffin cells. When applied to the data of three published studies, the model predicts that this factor is virtually non-functional in autistic individuals (Table 1).

Before the biological nature of this factor is discussed, it should be noted that the parameter values obtained for each of the three published studies were virtually the same (Table 1). This underlying consistency of the data is not trivial, since Mulder et al. [39] have suggested that their autistic distribution may be bimodal and thus qualitatively different from the control (normal) distribution, whereas Coutinho et al. [40] have reported a clearly unimodal autistic distribution that so overlapped with the control distribution that their means were not statistically significant. It should also be noted that initially *γ *was allowed to vary from zero to one, but the numerical optimization based on the published data narrowed this range down to approximately 0.8 – 1.0 (Table 1). This agrees well with actual experimental data. An early study has approximated the dog's *γ *as 0.99 and shown that the 5-HT clearance by the lungs varies from 0.80 to 0.98 [60]. The mean human *γ *may be somewhat smaller, because the rate of 5-HT release by gut enterochromaffin cells has been predicted to be around 3000 ng/min [53] and the arterial flow of free 5-HT has been estimated to be around 210 ng/min [48,53,70]. This suggests that, in humans, approximately 93% of free 5-HT is cleared in one circulation and, therefore, the value of *γ *is close to 0.93. The model predicted similar *γ *distributions in normal and autistic groups, supporting the hypothesis that the frequencies of SERT and MAO polymorphisms in normal and autistic groups may be the same.

The most significant result is that the factor that regulates 5-HT release from EC cells (represented by the parameter *α*) appears to be virtually non-functional in autistic individuals (Table 1). What is the biological nature of *α*? Evidence suggests that EC cells may express 5-HT_3_, 5-HT_4 _and 5-HT_1A _receptors [55,88-90] and that they may also express 5-HT_2 _receptors [89]. Some of these receptors appear to be involved in the autoregulation of 5-HT release [89,90]. While one report has failed to find 5-HT_3 _and 5-HT_4 _receptor mRNAs in cultured EC cells [91], the regulation of 5-HT release from EC cells may also be indirect, by way of enteric neurons. These neurons are known to express various 5-HT receptors [54,55,92,93] and can control 5-HT release from EC cells by acting on their cholinergic and other receptors [88,94-96].

The model is based on a negative feedback loop. It has been shown that such negative feedback may be mediated by 5-HT_4 _receptors expressed by EC cells and that this negative feedback appears to dominate over the positive feedback mediated by 5-HT_3 _receptors [89,90]. A recent study has suggested that under normal circumstances (as opposed to conditions such as carcinoid tumors) the concentration of endogenous 5-HT may not be high enough to activate 5-HT_4 _receptors and alter the 5-HT release from EC cells [89]. At least superficially, this mirrors recent findings in the brain, where 5-HT_1A _and 5-HT_1B _receptors, long assumed to act as autoreceptors, may not actually be activated by extracellular 5-HT unless its concentration reaches excessive levels [51]. Since precise measurements of 5-HT release in the gut and the brain are difficult, it is more likely that these receptors do control 5-HT release under normal circumstances, but that their effect on 5-HT release is more subtle than we expect. The model's small value of *α *appears to predict such subtle regulation.

Can 5-HT_4 _receptors be involved in autism? One agonist used to study the effects of 5-HT_4 _receptors on the 5-HT release from EC cells has been 5-methoxytryptamine (5-MT) [89,90], which has high affinity for these receptors [57]. While 5-MT has been reported to inhibit the 5-HT release from EC cells, subcutaneous 5-MT injections in pregnant rats produces pups with autistic-like symptoms [97] and subcutaneous 5-MT injections in pregnant mice may lead to an autistic-like disruption of cortical columns in the pups [11,81]. Normal brain development may be altered if brain 5-HT_4 _receptors are compromised, because these receptors appear to be expressed in the marginal zone of the adult human brain [98] and, therefore, may also be expressed in Cajal-Retzius cells of the developing brain. It has been recently shown that an abnormal serotonergic input to Cajal-Retzius cells during development may lead to autistic-like cortical abnormalities [81]. Interestingly, the expression of the 5-HT_4 _receptor is very low in the cerebral cortex of the guinea pig [99], suggesting that this receptor may play a specific role in the primate brain. Generally, we are only beginning to understand the role of the 5-HT_4 _in brain development, because the human 5-HT_4 _receptor gene consists of at least 38 exons and at least eight C-terminal splice variants of the human 5-HT_4 _receptor have been described [57].

Other 5-HT receptors, as well as other mechanisms, may be involved both in the regulation of 5-HT release from the gut and in brain development. For example, 5-HT_1A _and 5-HT_2 _receptors have been implicated in autism [31,100-102]. As already discussed, these receptors can also regulate the 5-HT release from EC cells. Moreover, 5-MT is a rather non-specific 5-HT receptor agonist [103] and appears to be co-localized with 5-HT in most brain neurons [104]. Therefore, some of its effects may be produced by its acting on a few types of 5-HT receptors at the same time, both in the gut and the brain.

The model assumes that the 5-HT clearance rate (*γ*) and the gain of 5-HT release (*α*) are independent. Generally, the expression of neurotransmitter receptors or their sensitivity can dynamically change depending on the availability of the neurotransmitter. For example, gut 5-HT_3 _receptors undergo structural and functional changes in SERT-knockout mice [105] and 5-HT_1A _receptors in the human brain have different affinities in individuals with different SERT polymorphic variants [106]. These and other related findings are likely to become indispensable for understanding the platelet hyperserotonemia of autism; unfortunately, too little information is currently available for quantitative modeling of these relationships.

Intriguingly, *α *may be represented by biological mechanisms other than 5-HT receptors. For example, adenosine and ATP may modulate the 5-HT release from human EC cells [107,108] and ATP also activates microglia in the brain [109]. A study, called by some researchers "the most important postmortem study of autism to date" [110], has found an abnormal activation of microglia in autistic brains [111].

It should be noted in conclusion that the mathematical framework of the model allows it to be modified so that it no longer depends on free 5-HT in the blood. In fact, one could conceivably build a model where 5-HT is released by EC cells, cleared by SERT-expressing cells locally, and where the remaining extracellular 5-HT acts on the mechanisms controlling 5-HT release from EC cells, without leaving the gut. Assuming *γ *now denotes the local clearance and *α *is the gain of the 5-HT release, one again may arrive at a system of equations similar to equations (1) and (2).

## Conclusion

The origin of autism is as much a conceptual problem as it is experimental. The theoretical approach introduced here brings together information on the "central" and "peripheral" 5-HT and offers new insights into early abnormalities of the developing autistic brain that may otherwise escape direct experimental detection.

## Methods

All symbolic and numerical calculations were done in Mathematica 5.0.0, 5.0.1, 5.1.0, or 5.1.1 (Wolfram Research, Inc.). Where the numerical minimization of the error function produced different sets of numerical values in different releases of Mathematica, the values that yielded the smallest error were used (for the purpose of this study, Mathematica 5.1.1 was superior to the earlier releases). The figures were generated in Mathematica and prepared for publication in Adobe Illustrator 10 or CS (Adobe Systems, Inc.).

## Competing interests

The author(s) declare that they have no competing interests.

## Authors' contributions

SJ conceived of and carried out the presented study.
